# The Design and Manufacturing Process of an Electrolyte-Free Liquid Metal Frequency-Reconfigurable Antenna

**DOI:** 10.3390/s21051793

**Published:** 2021-03-05

**Authors:** Peng Qin, Lei Wang, Tian-Ying Liu, Qian-Yu Wang, Jun-Heng Fu, Guan-Long Huang, Lin Gui, Jing Liu, Zhong-Shan Deng

**Affiliations:** 1 CAS Key Laboratory of Cryogenics, Technical Institute of Physics and Chemistry, Chinese Academy of Sciences, Beijing 100190, China; qinpeng17@mails.ucas.edu.cn (P.Q.); wanglei_820425@163.com (L.W.); liutianying17@mails.ucas.edu.cn (T.-Y.L.); wangqianyu19@mails.ucas.edu.cn (Q.-Y.W.); fujunheng17@mails.ucas.edu.cn (J.-H.F.); lingui@mail.ipc.ac.cn (L.G.); jliu@mail.ipc.ac.cn (J.L.); 2 School of Future Technology, University of Chinese Academy of Sciences, Beijing 100049, China; 3 The College of Electronics and Information Engineering, Shenzhen University, Shenzhen 518060, China; guanlong.huang@ieee.org

**Keywords:** antioxidation, frequency-reconfiguration, liquid metal, temperature sensor, antenna sensor, thermal expansion, electrolyte-free

## Abstract

This communication provides an integrated process route of smelting gallium-based liquid metal (GBLM) in a high vacuum, and injecting GBLM into the antenna channel in high-pressure protective gas, which avoids the oxidation of GBLM during smelting and filling. Then, a frequency-reconfigurable antenna, utilizing the thermal expansion characteristic of GBLM, is proposed. To drive GBLM into an air-proof space, the thermal expansion characteristics of GBLM are required. The dimensions of the radiating element of the liquid metal antenna can be adjusted at different temperatures, resulting in the reconfigurability of the operating frequency. To validate the proposed concept, an *L*-band antenna prototype was fabricated and measured. Experimental results demonstrate that the GBLM in the antenna was well filled, and the GBLM was not oxidized. Due to the GBLM being in an air-proof channel, the designed liquid metal antenna without electrolytes could be used in an air environment for a long time. The antenna is able to achieve an effective bandwidth of over 1.25–2.00 GHz between 25 °C and 100 °C. The maximum radiation efficiency and gain in the tunable range are 94% and 2.9 dBi, respectively. The designed antenna also provides a new approach to the fabrication of a temperature sensor that detects temperature in some situations that are challenging for conventional temperature sensing technology.

## 1. Introduction

In recent years, the reconfigurable antenna has emerged as a promising candidate to face the challenges and requirements of high gain, broadband and multifunction in advanced communication systems. Generally, switching components such as radio frequency microelectromechanical systems (RF MEMS) [1], varactor diodes [2] and p-type intrinsic n-type (PIN) diodes [3] are frequently applied to ensure sensitive control of antennas’ reconfigurable performance. More and more radio frequency (RF) switches are used in antennas to seek better reconfigurable effects, while the complex auxiliary circuits and nonlinear effects remain inevitable. Moreover, some possible challenges, like constrained tuning and poor harmonics, require more strategies and solutions. Currently, ionic solutions [4], liquid crystals [5] and liquid metals, for example, provide new methods for reconfiguration. Among them, the outstanding characteristics of fluidity, electrical conductivity and deformability of liquid metal (LM) promise to become especially important in the field of reconfigurable electronic devices. Up to now, the potential of LM-based antennas has been demonstrated via pattern-, frequency- and polarization-reconfigurable prototypes [6,7,8,9,10,11,12,13,14,15,16,17,18,19,20,21,22,23], in which the LM mainly performs as a deformable structure for shape-patterning or switching in the radiating elements.

As a conventional LM, mercury has been applied to antennas since 1939 [6,7,8,9,10]. Although mercury is not easily oxidized at room temperature, it is volatile and highly toxic. The effects of mercury exposure can be very severe and subtle. At present, the mercury ban is a global trend. Gallium-based LM (GBLM) is a good substitute for mercury due to its non-volatile and non-toxic properties [11,12,13,14,15,16,17,18,19,20,21,22,23]. However, the oxidation of gallium greatly limits the antenna application of GBLM, since gallium oxide is facile to stick to the surface of the cavity [9,24,25]. Under the effects of surface tension and oxidation, mercury and GBLM fill, and withdraw from, microchannels, showing different behaviors [24]. GBLM requires more pressure to move into the microchannel than mercury. When the pressure is removed, GBLM cannot withdraw from the microchannel like mercury does, but requires HCl solution to eliminate gallium oxide. Compared with mercury, it is more difficult to control the flow of GBLM in the microchannel. Only in a glove box, where the oxygen concentration is less than 1 ppm, can GBLM maintain its unoxidized morphology [25]. Due to the low oxygen concentration threshold, the reaction between GBLM and oxygen occurs very easily. In [9], scientists tried to make Galinstan antennas in a glove box with an oxygen concentration of less than 1 ppm, but the antenna could only be used in the glove box and, once removed, it was ineffective. Additionally, the radiation efficiency of the antenna may be reduced since the electrical conductivity of gallium oxide (5 × 10^−4^ S/m [26]) is much lower than that of GBLM. Almost all existing research works on GBLM antennas either ignore the trouble of oxidation or add acid/alkaline electrolytes [12,13,14,15,16,17,18,19,20,21,22,23]. The electrolytes used in the deoxidation process produce three serious problems. Firstly, GBLM and acid/alkaline solutions react with and consume one another, so the system cannot coexist. Secondly, through the chemical reaction, the system (especially after the electrolysis reaction) generates bubbles, and multiple fluids can easily disconnect the GBLM. Thirdly, the presence of electrolytes will reduce antenna efficiency [27]. Therefore, avoiding oxidation or deoxidation with an electrolyte-free method is crucial for a GBLM reconfigurable antenna, both in terms of physical fabrication and practical application.

This paper presents a novel idea to drive GBLM into a confined space, and proposes a new type of GBLM-based reconfigurable antenna. Herein, by adopting the integrated manufacturing process of metal smelting under high vacuum, and GBLM infusion under a high-pressure shielding-gas atmosphere, an electrolyte-free antenna radiating element nicely filled with non-oxidized GBLM is obtained. In addition, in order to drive GBLM into enclosed spaces, a thermally controlled strategy is applied to generate the volumetric expansion of GBLM. Thus, non-contact and accurate control for the height of the metallic cylinder in the capillary of the antenna can be realized. In short, this work proposes a new idea of a thermally reconfigurable control method, and a new electrolyte-free manufacturing process for liquid metal antennas, which may pave a possible way toward the simplification of reconfigurable antennas in the future.

## 2. Antenna Design

### 2.1. Material and Structure

**Figure 1:**
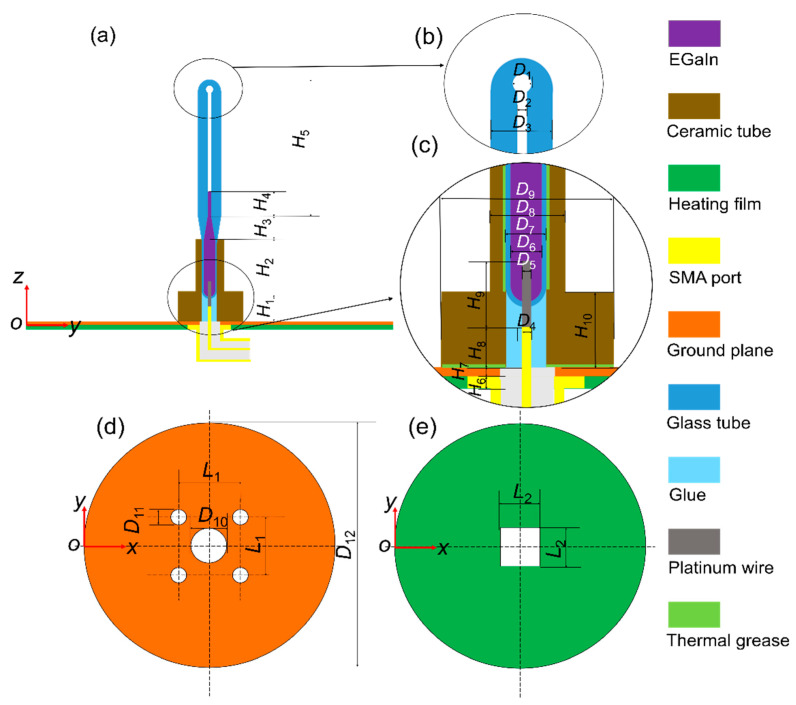
Configuration of the proposed monopole antenna. (**a**) Front view. (**b**) Top zoom. (**c**) Bottom zoom. (**d**) Ground plane. (**e**) Heating film.

Figure 1 shows the proposed monopole antenna structure, which consists of five main parts: a SubMiniature version A (SMA) connector, a heating film, a ground plane, an antenna radiating element and a ceramic tube. Enlarged views of the top and bottom parts of the radiating element are shown in Figure 1b,c. The shell of the antenna radiating element is made of quartz glass (relative permittivity: *ε_r_* = 3.7, the tangent of dielectric loss angle: tan *δ* = 0.00011, at 1 GHz [28]). The bottom part of the radiating element is a temperature-sensitive bulb filled with EGaIn (a kind of GBLM with the composition of 75.5% gallium and 24.5% indium, electrical conductivity *σ* = 3.46 × 10^6^ S/m) to make a reconfigurable wire. The platinum wire (*δ* = 9.43 × 10^7^ S/m) at the bottom of the radiating element is welded to the inner conductor of the SMA connector, which could be used to connect the feeder.

The bottom views of the ground plane and heating film are shown in Figure 1d,e, respectively. The heating wire of the heating film is wrapped in rubber, and its temperature is adjusted through a temperature control system. The heating film and the ground plane form a heating platform. The heating film, placed under the ground plane, is used to avoid the influence of the metal heating wire on the antenna radiating element. Ceramic tubes and thermal grease are applied to ensure that the heat on the heating platform can be effectively transferred to the EGaIn. The temperature change of the liquid metal is obtained by adjusting the electric current loads of the heating film. A calibrated thermocouple with ±0.5 °C accuracy was applied to detect the temperature of the liquid metal.

### 2.2. Principles of Reconstruction

The degree of liquid expansion is usually quantified by the cubic expansion coefficient *γ*, which is defined by:

(1) $$\gamma =\frac{1}{V_{0}}{\left(\frac{\partial V}{\partial T}\right)}_{P}\;$$

where *V* is the volume, *T* is the temperature, *P* is the pressure and *V*_0_ is the initial volume at the initial temperature *T*_0_.

EGaIn usually remains in liquid state from 15.5 °C to 2000 °C [29] and has the characteristics of cold shrinkage and thermal expansion. Its liquid-phase temperature range is much larger than that of mercury, from −38.8 °C to 356.7 °C [30]. The thermal expansion coefficient of quartz glass is 5.5 × 10^−7^ 1/K [31], which can be ignored compared to the thermal expansion coefficient of GBLM [32].

Hence, *V*, the volume of liquid metal, can be expressed by the dimensions marked in Figure 1, i.e., *D*_2_, *D*_6_, *H*_2_, *H*_3_ and *H*_4_:

(2) $$V=\frac{\pi }{12}\left(D_{6}^{3}+3D_{6}^{2}H_{2}+D_{2}^{2}H_{3}+D_{6}^{2}H_{3}+D_{2}D_{6}H_{3}+3D_{2}^{2}H_{4}\right)=\frac{\pi }{4}C+\frac{\pi }{4}D_{2}^{2}H_{4}$$

where *C* is a constant value during temperature variation, and it can be expressed as:

(3) $$C=\frac{1}{3}\left(D_{6}^{3}+3D_{6}^{2}H_{2}+D_{2}^{2}H_{3}+D_{6}^{2}H_{3}+D_{2}D_{6}H_{3}\right)$$

According to (1), the volume of liquid metal can be rewritten as (4) when the temperature increases by ∆*T*:

(4) $$V=V_{0}+\Delta V=V_{0}\left(1+\gamma \Delta T\right)$$

Additionally, the relationship between *H*_4_ and ∆*T* can be derived from (2) and (4):

(5) $$H_{4}=H_{40}+\frac{C+D_{2}^{2}}{D_{2}^{2}}\gamma \Delta T$$

where *H*_40_ is the initial length of *H*_4_ at the initial temperature *T*_0_. After the geometric structure is determined, *T* is the only variable of *H*_4_. As the length *L* (*L* = *H*_1_ + *H*_2_ + *H*_3_ + *H*_4_) of the radiating element of the monopole antenna generally should satisfy the condition of *L* = *λ*/4, the resonant frequency of the antenna can be changed by controlling the temperature.

### 2.3. Parametric Analysis

**Figure 2:**
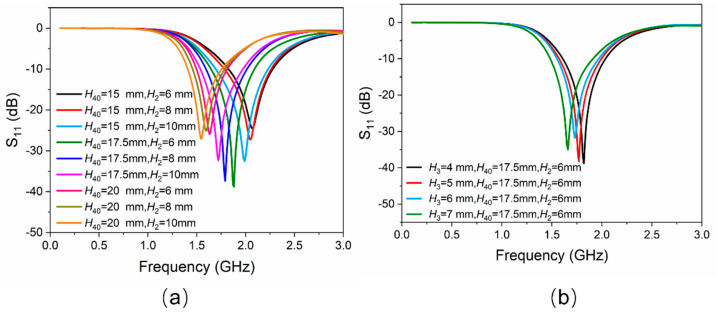
(**a**) Simulated results of |S_11_| versus the variation of EGaIn column *H*_40_ and the height of the cylinder of temperature-sensitive bulb *H*_2_. (**b**) Simulated results of |S_11_| versus the variation of the height of the cone of temperature-sensitive bulb *H*_3_.

The working frequency band of the antenna proposed in this work is designed in the *L*-band (1–2 GHz). As mentioned above, the lengths of EGaIn mainly determine the working frequencies of the proposed antenna. All the initial geometric dimensions of the antenna structure (for example, *H*_2_, *H*_3_, *H*_40_) need to be uniquely confirmed. A full-wave electromagnetic high-frequency structure simulator (HFSS) was used to simulate the proposed antenna in this communication. The entire antenna, except the SMA connector, was considered as the simulation model. Figure 2a shows a clear change in reflection coefficient values (represented by S_11_), as *H*_40_ and *H*_2_ changes. It can be seen that the smaller *H*_40_ is, the higher the operating frequency of the antenna and the lower the reflection coefficient would be. However, the initial value of *H*_40_ should be large enough so that EGaIn would not flow back into the temperature-sensitive bulb when the temperature drops. Therefore, trade-off values should be chosen, while taking the highest working frequency of 2 GHz into consideration. In this work, the initial lengths of *H*_40_ and *H*_2_ were chosen to be 17.5 mm and 6 mm, respectively. Figure 2b shows a little change in reflection coefficient values as *H*_3_ changes. The length of *H*_3_ is finally assigned to be 7.0 mm with a lower reflection coefficient value. By using this method of controlling variables, other dimensions can also be optimized similarly. The optimized dimensions of the proposed antenna shown in Figure 1 are given in Table 1.

## 3. Fabrication and Measurement

### 3.1. Fabrication Process

Initially, in a glove box with nitrogen protection, an attempt was made to inject GBLM through a syringe into an open glass tube. However, the antenna failed after being removed from the glove box because the GBLM was still oxidized. Later, a mechanical pump was used to extract air from the semi-closed glass tube. The prepared liquid metal was filled into the glass tube by the pressure difference between the inside and outside of the glass tube. The fabricated antenna still had the problem that GBLM had been oxidated and incompletely filled. Through many previous failed experiments, two important pieces of information were confirmed. Firstly, the GBLM would be partially oxidized rapidly (when exposed to air) before it was applied in antenna manufacturing. Secondly, it was difficult to completely fill the channel using both manual perfusion and pressure perfusion with a mechanical pump. Therefore, an integrated solution for preparing GBLM under high-vacuum conditions and filling GBLM under high-pressure conditions was proposed.

**Figure 3:**
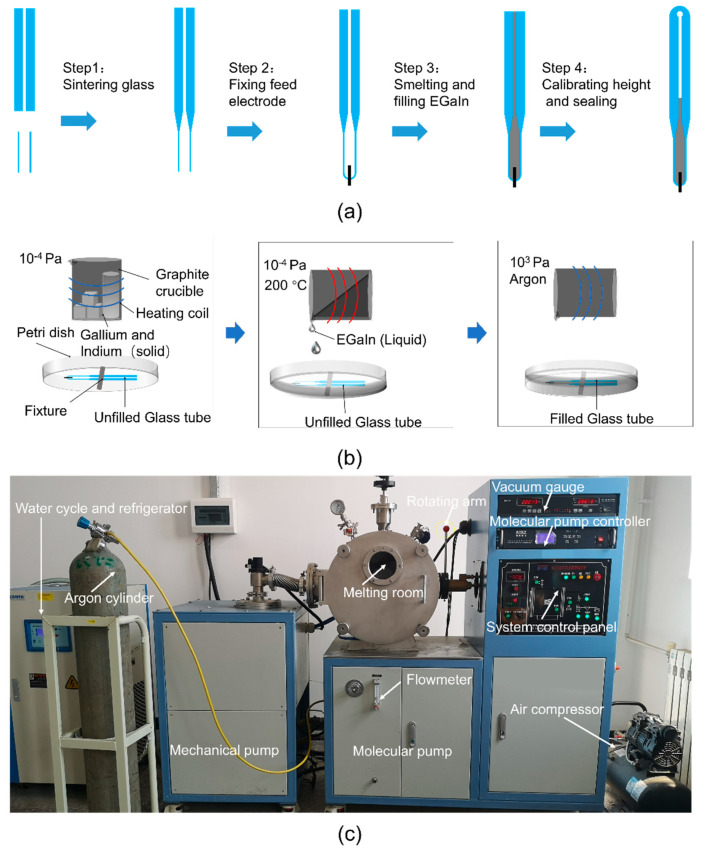
(**a**) The manufacturing process of the antenna radiating element. (**b**) The smelting and filling processes of EGaIn (Step 3). (**c**) The smelting and filling equipment of EGaIn (Step 3).

The final schematic manufacturing process of the proposed liquid metal antenna is shown in Figure 3a. Firstly, the glass tube used as the temperature-sensitive bulb, and the capillary tube, were sintered and connected. Secondly, the platinum feeding electrode and the glass tube were sintered together. The feeding electrode was made of platinum wire because platinum is not easily oxidized and has high conductivity. Thirdly, EGaIn was prepared and filled into the glass tube under a vacuum environment. The relationship between temperatures and heights was properly marked. The temperature-sensitive bulb was heated so that the excess EGaIn was discharged. Meanwhile, the top of the glass tube was sintered and sealed. Steps 1, 2 and 4 are common measures for the manufacture of thermometers, which were entrusted to Wuqiang Huayang Instrument Co., Ltd., China. Step 3 will be described in more detail below.

**Figure 4:**
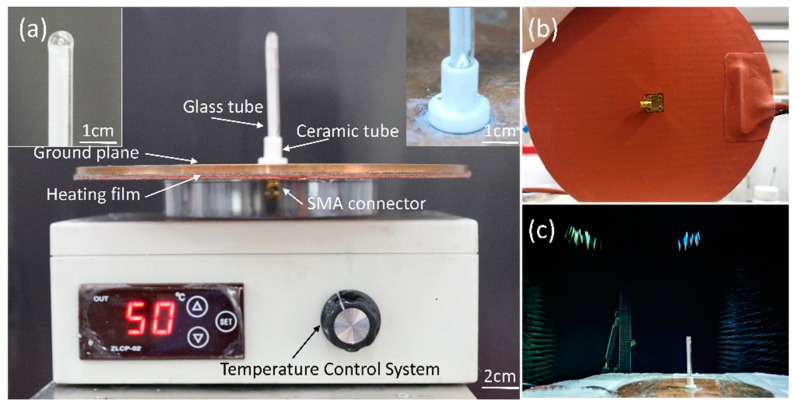
Fabricated antenna prototype. (**a**) Front view. The left illustration is the enlarged view of the top of the glass tube. The right illustration is the enlarged view of the bottom of the glass tube. (**b**) Bottom view. (**c**) Measurement setup of the liquid metal reconfigurable antenna in an anechoic chamber.

As shown in Figure 3b, the fusion and infusion processes of EGaIn (Step 3) were carried out in a two-stage vacuum system to avoid oxidation. This two-stage vacuum system is a miniaturized vacuum induction smelting furnace customized by Shanghai Mengting Instrument Co., shanghai, China. As shown in Figure 3c, it contains two-stage pumps (a mechanical pump and a molecular pump), a smelting chamber (volume approximately 0.1 m^3^), an induction coil, cooling water, a refrigerator, an air compressor, a vacuum gauge and a rotating arm, among other things. Firstly, the metal elements were weighed in proportion and placed in a graphite crucible (inner diameter 44 mm, height 100 mm, made by Guanzhi New Material Technology Co., Guangzhou, China). Graphite crucibles were used here, instead of ceramic crucibles, because graphite crucibles are reductive and can better protect metals from oxidation. The graphite crucible containing the metal elements for smelting GBLM was placed in the induction coil, and the petri dish with the empty glass tube was put in a suitable position below the graphite crucible. After that, the mechanical pump was turned on for half an hour to reach a low vacuum (10 Pa) in the melting chamber, and then the molecular pump was turned on for two hours to reach a high vacuum (10^−4^ Pa). Subsequently, under a high vacuum, EGaIn was prepared at 200 °C, and poured into a petri dish by the rotating arm, submerging the unfilled glass tube. Finally, 300 kPa of argon was filled into the two-stage vacuum system. EGaIn was forced into the empty glass tube under a great pressure difference. This integrated process of smelting EGaIn in a high vacuum, and injecting EGaIn in a high-pressure protective gas, ensured that a fully filled and non-oxidized antenna radiating element was obtained. The proposed antenna, after the assembly, is shown in Figure 4.

During the fabrication process in this work, the EGaIn did not become oxidized when it was smelted and filled into the antenna. Moreover, the non-contact thermal expansion strategy utilized here could enable the EGaIn to be completely sealed in a vacuum container, eliminating the necessity for electrolytes. The designed antenna can be applied in the air for a long time without worrying about oxidation.

### 3.2. Results and Discussion

The Keysight Technologies N5247A vector network analyzer (VNA) was used to measure the antenna’s reflection coefficient, and its radiation characteristics were tested in a microwave anechoic chamber, as shown in Figure 4c.

**Figure 5:**
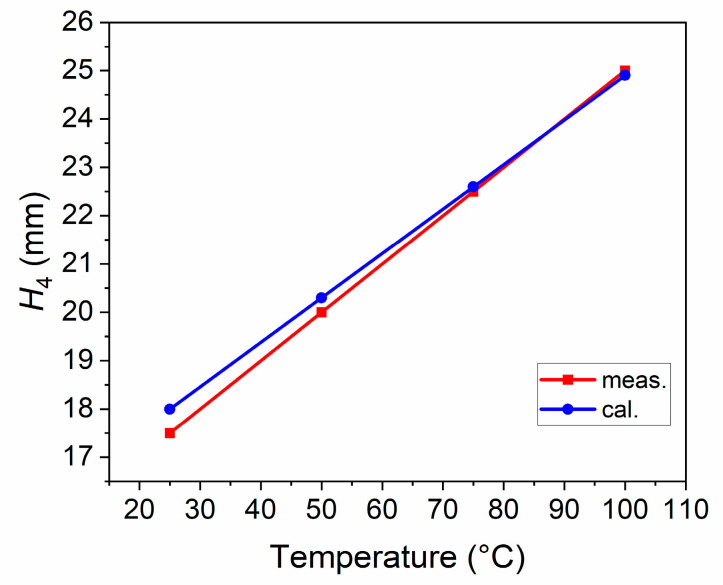
The lengths of *H*_4_ at different temperatures.

The cubic expansion coefficients of EGaIn at 40–100 °C were measured, as shown in Table 2. In addition, the data in Table 1 and Table 2 were brought into (1)–(5), and the lengths of *H*_4_ at different temperatures were calculated and compared with the measurement results, as shown in Figure 5. As can be seen, the length of *H*_4_ increases linearly with temperature, and the measurement and calculation results are consistent. These modest differences could be traced to the failure of eliminating the expansion effect of the quartz tube when measuring the cubic expansion coefficients of EGaIn, and inaccurate reading when measuring the length of *H*_4_.

**Figure 6:**
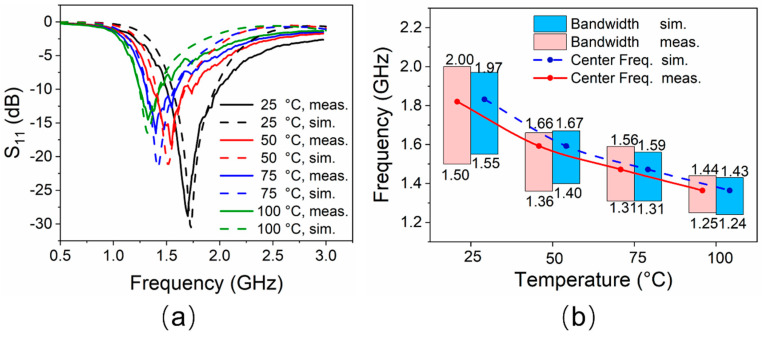
(**a**) Reflection coefficients of the antenna at different temperatures. (**b**) Impedance bandwidths performance of the antenna at different temperatures.

The reflection coefficients of the proposed antenna at different temperature states are shown in Figure 6a, and the relationship between impedance bandwidth and temperature is shown in Figure 6b. Under ambient temperature (25 °C), simulated and measured bandwidths with a reflection coefficient below −10 dB are 24.4% (1.55–1.97 GHz, resonated at 1.72 GHz) and 29.4% (1.50–2.00 GHz, resonated at 1.70 GHz), respectively. As the temperature gradually rises from 25 °C to 100 °C, EGaIn in the glass tube expands. The measured length of *H*_4_ increases from 17.5 mm to 25 mm linearly. Hence, the resonant frequency of the antenna decreases. The simulated frequency range with |S_11_| < −10 dB reduces to 1.24–1.43 GHz (resonated at 1.32 GHz), while the corresponding measured results decrease to 1.25–1.44 GHz (resonated at 1.32 GHz) by degrees.

This frequency reconstruction process would be expected to be continuously changing since the temperature changes are successive. The working frequency band basically covers the *L*-band, and the minimum reflection coefficient is better than −15 dB for all the cases. The simulated results are in good agreement with the measured ones. The slight difference is mainly observed in the lower reflection coefficient obtained by the measurement, which may be due to manufacturing errors and reflections from the environment.

**Figure 7:**
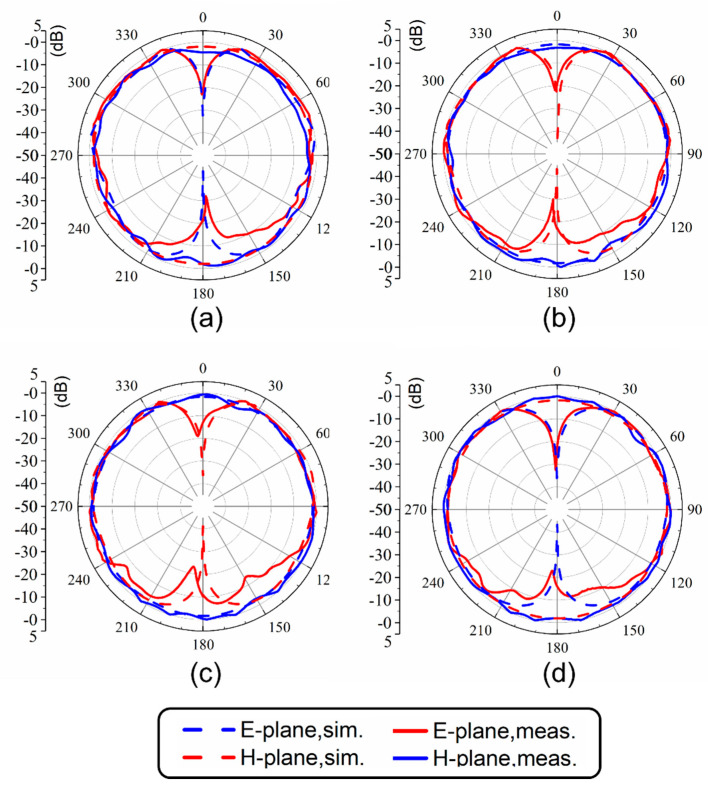
Radiation patterns at (**a**) 1.7 GHz (25 °C), (**b**) 1.51 GHz (50 °C), (**c**) 1.41 GHz (75 °C) and (**d**) 1.32 GHz (100 °C).

**Figure 8:**
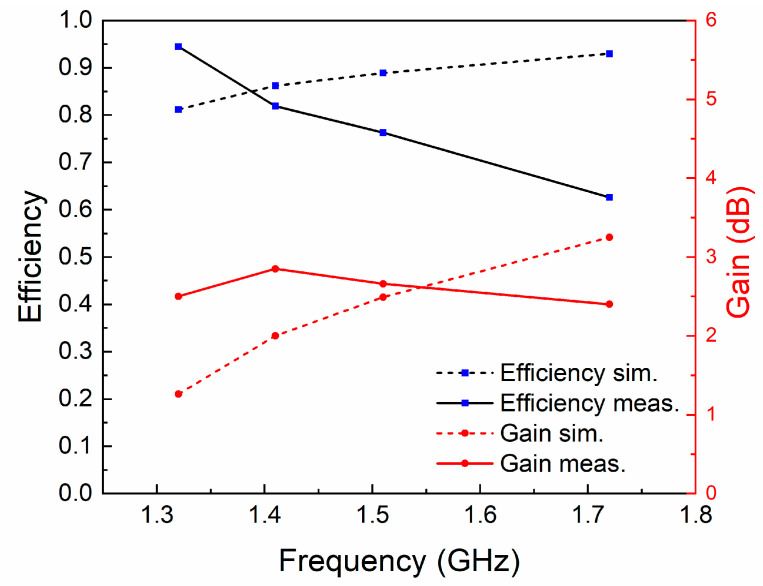
The radiation efficiencies and gains at different resonant frequencies.

The normalized radiation patterns of the *E*-plane (X-Z plane) and *H*-plane (Y-Z plane) at four different temperatures and resonant frequencies of the antenna are shown in Figure 7. Similarly to simulated results, this antenna shows typical radiation patterns for a classical monopole antenna with a stable omnidirectional radiation on the *H*-plane and a bidirectional radiation on the *E*-plane. Furthermore, radiation efficiencies in different cases were calculated using the maximum gain and directivity. As shown in Figure 8, the maximum radiation efficiency and gain are 94% and 2.9 dBi, respectively, in the tunable range. There are differences in gain and efficiency trends between the simulations and measurements, because the simulations represent ideal conditions, which are not completely consistent with the actual conditions. The measurement results show that the loss of the antenna increases as the frequency increases, resulting in a decrease in efficiency and gain. The loss comes from dimensional errors, dielectrics, cables and interfaces.

The prototype antenna was fabricated to verify the feasibility of the thermal expansion-based strategy, currently with a tuning range of 1.25–2.00 GHz, but this is not the limit for the thermal-expansion-based antenna. For example, higher operating frequencies can be obtained by reducing the geometric size, especially by tuning *H*_1_, *H*_2_, *H*_3_, *H*_4_ and *D*_2_, or lowering the operating temperature, unless the liquid–solid phase transition occurs. When the geometry is determined, the lowest working frequency is determined by the maximum operating temperature. Considering the heat resistance of common materials and the safety of measurements, as a prototyped antenna, its operating temperature range in the aforementioned measurements is only 25–100 °C. Under the geometric dimensions shown in Table 1, the trend in Figure 6 is that when the length of *H*_4_ increases by 10 mm every time, the temperature rises by 100 °C. The increased operating temperature can further reduce the resonance frequency. In short, the smaller geometric size and wider operating temperature range can greatly broaden the working bandwidth of the antennas, based on the proposed control method. Moreover, the antenna proposed in this study can also serve as a temperature sensor for high-temperature measurement, since the boiling point of liquid metal is extremely high. When working as a temperature sensor, the heat source to be measured replaces the heating platform. The designed antenna will have both temperature sensing and signal transmission functions. In measuring high temperatures, the major concern is the heat resistance of the antenna materials. It is necessary to replace the organic glue in this study with high-temperature-resistant inorganic glue (heat resistance temperature can exceed 1300 °C [33]) and replace the Polytetrafluoroethylene radio frequency connector with the ceramic radio frequency connector (heat resistance temperature can exceed 600 °C [34]).

Several LM frequency-reconfigurable antennas, and traditional frequency-reconfigurable antennas, are listed in Table 3 for comparison. In [1], only two discrete frequencies can be reconfigured. Gains are not given in [3,11]; furthermore, efficiencies are not given in [2,3,9,11]. The tuning range is relatively small in [11] and the antenna needs an external force to achieve reconfigurability. The tuning range is broad in [9]. Since the oxidation problem of Galinstan was not solved well, highly toxic mercury was used instead of Galinstan in [9]. The principles of continuous electrowetting (CEW) and electrically controlled capillarity (ECC) are used to drive LM in [12,13], respectively. In [13], the peak efficiency is 70%, which is lower than the levels seen in this work, because the NaOH solution is used as a conductive liquid in the bias circuit and to eliminate gallium oxide in [12,13]. Compared with the above work, the antenna proposed in this communication is safer and more convenient to apply, since the oxidation problem is avoided without use of another protective device.

Nevertheless, the speed of the antenna’s response to temperature is expected to be improved. During the tests, it took 20–30 s to obtain a temperature difference of 25 °C. In other words, the temperature change rate is about 1 °C/s. In fact, a long reconfiguration time is a common problem for antennas using liquid metal through fluid flow, and the thermal-expansion-based actuation is no exception. Whether using external force methods [11], pumping methods [9], CEW [12] or ECC [13], the speed of liquid metal antennas is currently not comparable to control methods using radio frequency switches [1,2,3]. On the other hand, heating the plane requires more power. In the future, heating methods with a high density such as laser heating may be used to accelerate the reconfiguration procedure of thermal-expansion-based liquid metal antennas.

## 4. Conclusions

A frequency-reconfigurable antenna based on the thermal expansion of GBLM has been investigated and verified by measurement. A continuous operating frequency range of 1.25–2.00 GHz is acquired via manipulating the temperature between 25 °C and 100 °C using EGaIn. The tunable working bandwidths cover the majority of the *L*-band. Under the conditions of four representative temperatures, the maximum radiation efficiency and gain in the tunable range are 94% and 2.9 dBi, respectively. Moreover, during the fabrication process in this work, the GBLM did not become oxidized when it was smelted and filled into the antenna because an integrated process route was proposed, in which GBLM was smelted in a high vacuum and injected into the antenna in a high-pressure protective gas. In brief, a thermal-control strategy is proposed in order to realize, for the first time, frequency reconfigurability of a monopole antenna via the thermal expansion of GBLM. This provides a promising solution for reconfigurable antennas based on liquid metal techniques. The designed antenna can be used as a temperature sensor in some situations that are challenging for conventional temperature sensing methods.

## Figures and Tables

**Table 1:** Dimensions of the Optimized Antenna (Unit: mm).

*H*_1_	*H*_2_	*H*_3_	*H*_40_	*H*_5_	*H*_6_
6.0	6.0	7.0	17.5	37.0	2.0
*H*_7_	*H*_8_	*H*_9_	*H*_10_	*D*_1_	*D*_2_
2.0	4.0	4.2	6.0	2.0	0.32
*D*_3_	*D*_4_	*D*_5_	*D*_6_	*D*_7_	*D*_8_
6.3	0.5	0.5	3.0	4.0	8.6
*D*_9_	*D*_10_	*D*_11_	*D*_12_	*L*_1_	*L*_2_
14.8	4.0	2.0	100.0	5.0	14.0

**Table 2:** The Cubic Expansion Coefficients of EGaIn.

**Temperature (°C)**	40	50	60	70	80	90	100
***γ* × 10^6^ (1/K)**	113.1	114.3	115.0	115.4	116.1	117.4	118.6

**Table 3:** Performance comparison between different antennas.

Ref.	Radiator	Methods of Reconstruction	Liquid Metal	Solution Environment	Reconstruction Speed	Control Circuit Power	Reconfigurable Characteristics (GHz)	Peak Gain (Mea.)	Peak Efficiency (Mea.)
[1]	Planar inverted F	RF-MEMS	None	None	Fast	Small	0.718 and 4.96	3.3 dBi	85%
[2]	Quasi-Yagi	Varactor diodes	None	None	Fast	Small	6.0–6.6	6.35 dBi	N/A
[3]	Planar inverted U	PIN diodes	None	None	Fast	Small	2.63–3.7	N/A	N/A
[9]	Monopole	Micro pump	Mercury	Teflon solution	16 s	Medium	1.29–5.17	2.5 dBi	N/A
[11]	Dipole	External force	EGaIn	None	N/A	None	1.91–1.99	N/A	≈90%
[12]	Slot	Continuous electrowetting	Galinstan	NaOH solution	0.9 mm/s	Medium	2.2–2.6	2 dBi	N/A
[13]	Monopole	Electrically controlled capillarity	EGaIn	NaOH solution	3.6 mm/s	Medium	0.66–3.4	3.4 dBi	70%
This work	Monopole	Thermal expansion	EGaIn	None	≈1 s/°C	High	1.25–2.00	2.9 dBi	94%

## Data Availability

Not applicable.
